# An overview of platforms for cloud based development

**DOI:** 10.1186/s40064-016-1688-5

**Published:** 2016-01-16

**Authors:** G. Fylaktopoulos, G. Goumas, M. Skolarikis, A. Sotiropoulos, I. Maglogiannis

**Affiliations:** B-Open S.A., Laskaratou 11A, Pylaia, 54250 Thessaloniki, Greece; Computing Systems Laboratory, National Technical University of Athens, Athens, Greece; GRNET S.A., Av. Mesogion 56, 11527 Athens, Greece; Department of Digital Systems, University of Piraeus, Piraeus, Greece

**Keywords:** Cloud computing, Integrated Development Environment (IDE), Code repositories, Software modeling, Orchestration tools

## Abstract

This paper provides an overview of the state of the art technologies for software development in cloud environments. The surveyed systems cover the whole spectrum of cloud-based development including integrated programming environments, code repositories, software modeling, composition and documentation tools, and application management and orchestration. In this work we evaluate the existing cloud development ecosystem based on a wide number of characteristics like applicability (e.g. programming and database technologies supported), productivity enhancement (e.g. editor capabilities, debugging tools), support for collaboration (e.g. repository functionality, version control) and post-development application hosting and we compare the surveyed systems. The conducted survey proves that software engineering in the cloud era has made its initial steps showing potential to provide concrete implementation and execution environments for cloud-based applications. However, a number of important challenges need to be addressed for this approach to be viable. These challenges are discussed in the article, while a conclusion is drawn that although several steps have been made, a compact and reliable solution does not yet exist.

## Background

Within the past few years, cloud computing has emerged as a dominant computing model in IT infrastructures, enabling flexible, ubiquitous, on-demand and cost-effective access to a wide pool of shared resources (Barroso et al. 2013). Leveraging its various service types, large and diverse user communities have adopted the cloud paradigm enjoying the following two key offerings: (a) low costs by releasing users from the burden to invest on hardware infrastructures and software licenses, and (b) reduced operational complexity, as organizations are able to focus on the quality of their products and services rather than on the management of complex IT systems (Armbrust et al. 2010; Buyya et al. 2009). Economy of scale enables additional decrease in total market costs, as numerous small-scale and typically underutilized data centers are replaced by larger infrastructures that target higher resource efficiency (Beloglazov et al. 2012). On the application side, large families of applications including desktop, business, entertainment (Simmhan et al. 2010; Schmidt 2012; Hobfeld et al. 2012) etc., have found their way to the cloud creating a demanding and fast evolving new ecosystem.

The proliferation of the cloud paradigm has created a strong trend to transfer traditional services and applications to the cloud. This trend includes, among numerous others, file hosting and gaming for home users, office applications for home and professional users and large, complex business applications for customer management, logistics and collaboration. Not surprisingly, software development environments consist a critical application domain that has also gained significant popularity through its “cloudified” versions. Transferring major services and applications to the cloud has created new demands for productive software development. Cloud concepts and technologies provide a valuable substrate to support software development environments “in the cloud, for the cloud” as they can easily provide an ample pool of compute resources for code development and testing, and code repositories to support developer collaboration, a key driving force to software productivity (Anselmo and Ledgard 2003; Wu et al. 2009).

Traditional software development employs a toolchain including a text editor, a compiler and possibly a debugger, and performance analyzer. To accelerate software development, this rather disjoint tool chain is incorporated in an Integrated Development Environment (IDE) (Kats et al. 2012). In order to further reduce time to market and development costs, software engineers heavily encourage reuse of existing software components in order to create new services. The adoption of software development models, based on existing components (Component-based application development) (Heineman and Councill 2001), inherently supports high adaptability and scalability and allows a faster way of constructing applications, as developers focus on basic functional components to create new, higher levels of services. Especially for non-experienced and skilled developers, the ability to develop new applications through components’ synthesis is of paramount importance.

To build a successful cloud programming environment the advantages and functionality of traditional, desktop-based IDEs need to be maintained and augmented with additional features and strengths. Powerful code editors with a rich set of functionalities (e.g. highlighting, autofill, etc.) are incorporated in web browsers. Compilation (Ansari et al. 2011) and testing execution are performed on cloud infrastructures and in several cases deployment can be supported by cloud providers. Clear advantages of cloud-based IDEs include: (a) the access to a very wide pool of programming tools that are maintained by the provider, thus relieving the developer from the burden to setup, configure and upgrade their programming environments, (b) the ability to develop software without the use of powerful local computers, since the frequently compute-intensive tasks of the compilation and testing are performed remotely in the cloud, and (c) the straightforward way to reuse code developed by other software engineers that share the same cloud environment. However, a cloud application development environment needs to go far beyond a powerful toolchain to implement, debug and test code. For large-scale software projects to be viable and profitable, significant attention needs to be paid to the pre-implementation, design phase with the incorporation of appropriate modeling tools, and to post-implementation tasks including documentation and maintenance. Such features are rarely incorporated in cloud-based application development environments in their entirety a fact that makes them lag behind their desktop-based counterparts. On the other hand, network latency can also be a limiting factor (Piao and Yan 2010), especially for small jobs, while security and privacy concerns are certainly valid for valuable intellectual property as is the case of software code (Zhu et al. 2010; Zissis and Lekkas 2012).

In this paper we perform a review on the current technologies for software development in cloud environments. We focus on a number of topics including integrated programming environments, code repositories, software modelling, composition and documentation tools, and application management and orchestration. Moreover, we evaluate the existing ecosystem based on a wide number of characteristics like applicability (e.g. programming and database technologies supported), productivity enhancement (e.g. editor capabilities, debugging tools), support for collaboration (e.g. repository functionality, version control) and post-development application hosting. Based on our review, we draw a number of interesting conclusions regarding the maturity of the state-of-the-art solutions and discuss challenges and open issues that can guide future developments in this fast evolving technological field.

The rest of the paper is organized as follows: In “Cloud architectural approaches and models” section, we describe the various cloud models, while in “Existing platforms and cloud programming environments” section, we review existing platforms and cloud programming environments. In “Comparisons” section, we provide a comparison of the most prominent systems and in “Challenges and open issues” section we discuss the remaining challenges and open issues. Finally, “Discussion and conclusions” section concludes the paper.

## Cloud architectural approaches and models

### Generic models and architectures

There are three fundamental models according to which cloud providers offer their services: Infrastructure-as-a-Service (IaaS), Platform-as-a-Service (PaaS) and Software-as-a-Service (SaaS) (Voorsluys et al. 2011). IaaS is the model in which the cloud provider offers physical infrastructure or more often virtual machines (VMs) to the clients/consumers. Clients have to install their own operating system, libraries and applications, while they are responsible for managing the system. In the PaaS model, cloud providers offer a computing platform, and the client utilizes specialized tools offered by the cloud provider for the specific platform, in order to build its own framework or application on top of that platform. The client is responsible only for the developed framework/application, while the cloud provider maintains the underlying platform. Finally, in the SaaS model, the provider offers remote access to a domain specific application and/or database, already built by the provider. The client is usually unaware of the underlying platform running the application and/or database.

Service-Oriented Architecture (SOA) (Thomas 2004) has been established as the de-facto standard architecture for web applications. SOA enables the merging of distributed systems or the deployment of new business services by utilizing existing services in a particular order, following a business process. SOA essentially is a collection of communicating services. This communication may involve simple message passing between two services, or the coordination of multiple services to implement one new activity. SOA is independent of the programming language used or the underlying operating system. SOA is based on three basic technologies: a language for the description of web services called WSDL (Web Services Description Language), a protocol that enables the exchange of messages XML called SOAP (SOAP 2007) and a protocol that enables the publication and discovery of services called UDDI (Universal Description, Discovery and Integration). What makes SOA model extremely useful is the capability of discovering services that match the application’s needs, the capability of negotiating terms of use and the accessibility from any location at any time.

Web services constitute the modern way of developing applications, especially when remote access to distributed functions is needed. A web service is a technology that allows applications to communicate independently of platform or programming language. A Web Service is the software interface that describes a collection of functions that can be accessed from the network using XML messages. Web services utilize XML to describe both data and operations on these data. A group of interacting services define a new web service.

In the last years, considerable effort is taking place in order to provide cloud software standardization that will lead to high quality cloud software creation. This effort has resulted in several prominent standards in the cloud world. For example, Open Virtualization Format (OVF 2014) is a prototype design to tackle the portability and installation issues on VMs in the Cloud. Cloud Infrastructure Management Interface (CIMI 2014) is a prototype that standardizes the interactions among Cloud environments in order to achieve interoperable management between cloud service providers and service consumers/clients. Open Cloud Computing Interface (OCCI 2010) is a RESTful protocol that operates as a front-end service for the Cloud provider internal management. OCCI describes APIs that allow Cloud providers to publish the offered services. It enables the development, monitoring and management of VMs and can be applied to any interaction with a virtual cloud resource. IEEE-P2301 (Guide for Cloud Portability and Interoperability Profiles-CPIP) is a guide whose purpose is to assist cloud computing vendors and users in developing, building, and using standards-based cloud computing products and services. Target is to increase the portability and the interoperability of produces applications. IEEE-P2302 (Standard for Intercloud Interoperability and Federation-SIIF) creates an economy amongst cloud providers that is transparent to users and applications, which provides for a dynamic infrastructure that can support evolving business models. Open Authorization Protocol (OAUTH 2006) is an open standard for authorization. It enables users to share their private resources (e.g. photos, videos, etc.) without exposing their credentials (i.e. username, password). OpenID (OID 2014) is an open standard and decentralized protocol that allows users to be authenticated by sites using a third party service, eliminating the need for webmasters to provide their own ad hoc system. This allows users to have one digital identity for many different web sites/applications. Overall, development environments for cloud-based applications constitute a rather complex and dynamic ecosystem including a diverse set of standards, tools, techniques and technologies that need to be properly integrated to provide a viable approach for the development of quality software in reduced time-to-market.

### Specific approaches

There exist a number of research projects that are trying to tackle the specific problems in the area of code development in the Cloud. Below, we present a brief overview of a few relevant ones: Cloud4SOA, ASCI, REMICS and MONDO.

*Cloud4SOA* project (CLOUD4SOA 2010) targets the semantic interoperability issues that exist in modern cloud infrastructures. It introduces a new user-centric approach for application development and deployment in the Cloud. The proposed technologies combine three basic computing paradigms: Cloud, Service-Oriented Architecture and lightweight semantics. Cloud4SOA resulting solution is a scalable approach for the interconnection of heterogeneous PaaS services from different cloud providers that share the particular technology. Design consists of cooperating models and software components that provide developers with important capabilities, like matchmaking, management, monitoring and migration of applications.

*Artifact*-*Centric Service Interoperation (*ASCI 2010) project’s objective is to reduce the effort and lead-time of designing, deploying, maintaining, and joining into environments that support service collaborations. This is achieved by developing a rich framework based on the notions of (a) interoperation hubs and (b) dynamic artifacts. Interoperation hubs enable flexible, scalable support for service collaborations in an open network, while dynamic artifacts provide an approach to modeling and deploying business processes to simplify the management of data and interaction between different services and organizations. ACSI project develops improved process mining research by generalizing it to handle data along with process.

*REuse and Migration of legacy applications to Interoperable Cloud Services* (REMICS 2010) project has developed an advanced model driven methodology and the corresponding tools for reuse and migration of legacy applications to interoperable Cloud services. Service Cloud paradigm stands for combination of cloud computing and Service Oriented Architecture (SOA) for development of Software as a Service (SaaS) systems.

*MONDO: Scalable Modeling and Model Management on the Cloud* (MONDO 2007) project tackles the problem of scalability in Model Driven Engineering (MDE) (Beydeda et al. 2005; Stahl et al. 2006; Kleppe et al. 2003) by developing theoretical basis to advance the state of the art in model querying and transformations tools. It also provides an open-source cloud-based platform for scalable modeling and efficient storage, indexing and retrieval of large models having millions of model elements. MDE is a software engineering methodology that tries to reduce the complexity of software systems by advancing models that focus on the essential complexity of systems as first-class artefacts of the software development process. In contrast to traditional software development methodologies where models are mainly used for communication and post-mortem documentation purposes, models in MDE are the main living and evolving artifacts from which concrete software development artifacts can be produced in an automated fashion through model-to-model and model-to-text transformation.

The aforementioned research projects designate a number of challenges that need to be addressed towards the design and implementation of concrete cloud-based software development infrastructures, including interoperability, reusability, maintainability and scalability. Clearly, the field is new and constantly evolving, and a number of novel features and capabilities need to be incorporated in existing platforms to adapt to the new cloud era.

## Existing platforms and cloud programming environments

**Fig. 1:**
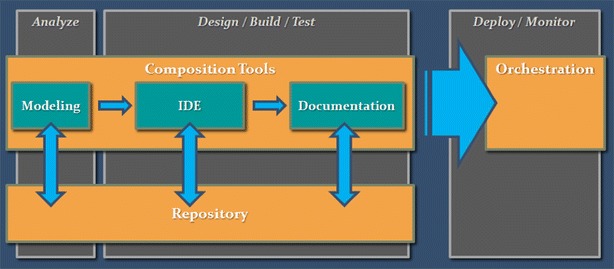
Application lifecycle and tools used in each phase

As already mentioned in “Background” section, the Cloud has quickly flourished and plenty platforms for Cloud software development, as well as Cloud programming environments are now available in the market. Depending on their operations, these platforms can be categorized as follows: (1) Programming Environments, (2) Repositories, (3) Modeling, Composition, (4) Processing, Documentation, Management and (5) Orchestration tools. A modern programming environment should include tools, which help the team fulfill all phases in the software development lifecycle. As described in Fig. 1, these may include modeling tools in the Analysis stage, IDEs for coding in the design/building phase, documentation tools, orchestration and management modules for the deployment and monitoring phase, and a repository used for version control during all phases.

In the following subsections we describe the State of the Art in each category. The surveyed tools operate mostly as stand alone applications with occasional interconnections mentioned in each case.

### Programming environments

Cloud programming environments are online web-based applications designed to offer development capabilities to developers. They usually consist of a source code editor, a number of compilers or interpreters depending on the programming language, a debugger and a project/solution viewer for managing the independent subcomponents. Apart from the default features and due to high competition, programming environments have evolved to include: connections to code repositories, collaboration features for code sharing, VMs for instant application deployment and even monitoring tools.

In Cloud’s early stages, programming environments offered only a web editor and a compiler. Developers could access their code using any web browser, but there were problems in terms of speed and reliability. Software like JavaWIDE (Jam et al. 2010) intended to provide a collaborative online IDE (Goldman et al. 2011) for schools and institutions with reduced availability of resources. Students would be able to write and test their JAVA code online, without losing time on platform installations. However, JavaWIDE did not manage to evolve and it slowly came to an end. This was also the result of Akshell (AKSHELL 2011), a Javascript IDE which tried to fill the gap of development and deployment in the cloud. Its features—unique for that time—were the ability to connect your code to your own PostgreSQL instance and its Git connectivity. Another tool whose life-cycle came to an end is Coderun (CODERUN). It supported Cloud development of Microsoft programming languages C#, .NET, ASP.NET, Silverlight and databases (SQL server). Its PHP, HTML, JavaScript and CSS plugins gave little support to the non-Microsoft development world.

Shortly, programming environments started to add features in order to stay competitive and gain more developers. Coderun managed to offer a huge variety or pre-installed compilers for many programming languages (C, C#, C++, Fortran, HTML, Javascript, Java, Objective C, Objective C++, Pascal, PHP, Python, Ruby, Visual Basic, ×86 Assembly). As it is a Cloud service, the programmer is able to access the source code from anywhere, using a mobile or desktop app. Though features like code completion and code sharing cannot be perceived as evolutionary, Compilr (COMPILR) success is coming from people who want to learn coding and not designing and deploying cloud applications. Its programming courses and the ability to “try what you just learnt” is the reason Lynda, a well-known online education company, acquired it in 2015. With the increasing usage of Javascript for web applications, jsFiddle (JSFIDDLE) appeared as a JavaScript sandbox or web playground. It is offered as a cloud service to developers who want to test their JavaScript code or offer it as an example to the community, often as a link to third party coding forums (like Stackoverflow). As it is a service specialized in JavaScript, it offers a plethora of well-known libraries that can be added directly in the tested code, as well as the ability to include external resources. Some other default features are the beautifier and pre-compiler, the HTML and CSS rendering and finally the code sharing.

However, even with the new features programming environments lacked deployment features in order to be considered as a full development solution. As a result, some of them started offering virtualization solutions for their users’ projects. Koding (KODING) for example, provided by Koding University, is offering a VM with SSH access and full documentation on how teams will be able to test, deploy and run their applications. It also provides tutorials on popular CMS and Database installations, trying to bridge the gap between the developer and the system administrator. It should be mentioned though that it is primarily used by students who want to start learning a programming language, like its predecessors, taking advantage of the Cloud SaaS architecture for educational purposes (Mehta and Gupta 2013).

As far as production programming environments are concerned, Cloud9 (CLOUD9) (Ciortea et al. 2010) has become the most popular Cloud programming environment, mostly because it expanded its service during mid-2014 and started offering full development solution for companies and not just tools for students or other institutions. Apart from the default development options (i.e. editors, compilers, and sharing tools), it offers specialized features for web development, like browser compatibility testing and live preview. However, the key point is that any application is placed under an Ubuntu Docker container, so the developers can install all the necessary software (i.e. web servers, CMS, mail servers etc.) with which their application can be deployed. On the other hand, this is a newly built technology, far from stable, not to mention that the whole installation process is not a “developer friendly” procedure and it needs a system administrator. Codenvy (CODENVY) is the main competitor of Cloud9. It has also changed its targeting from developers to DevOps, simplifying the way VMs are offered and installed through the Docker containers. The development kit contains a variety of programming environments, editors, databases and plugins. Codenvy is based on Eclipse Che cloud IDE (eclipse.org/che**)**, an open source IDE and SDK, which is can be easily extended. Projects are stored in workspaces that can be managed by DevOps, in order to gain code parity and security among teams and team members. DevOps are also responsible for the configuration and deployment of the applications through the platform’s built in tools. Codeanywhere (CA) has focused on providing a collaboration platform to developers. Sharing code, projects, files, folders or even whole environments are some of its collaborative features. Developers are able to view in real time the changes being made in their shared code by fellow developers and even lock parts of the code or manage the general access and change rights of parts of their project. The solution offered for deployment and installation is called DevBox, a private development environment with certain storage and memory running on OpenVZ (Docker will also be available soon as noted in their website).

Finally, Orion (ORION) is an open source project under Eclipse. It started as a Cloud IDE specifically focused on web development using JavaScript, CSS and HTML. Orionhub is the Cloud service using the Orion IDE, much like Codenvy uses Eclipse Che. However, due to awkward policy issues (e.g. no backup plan offered, code/accounts deleted due to inactivity), it cannot be considered as a viable solution for development teams who want to migrate to the cloud.

### Repositories

Cloud repositories are web-hosting facilities that leverage the strengths of well-known version control systems such as Git (GIT) (Lawrance and Jung 2013), Subversion (SUBVERSION) and Mercurial (MERCURIAL). In addition, they often support services and tools, including bug tracking, release management, mailing lists, and wiki-based documentation.

SourceForge (SF) was one of the first to offer a web-based source code repository that brings collaborators together and helps projects get developed, downloaded, reviewed, and published. It was also one of the first to offer this service for free to open source projects.

Apart from the version control system they are using, cloud repositories are distinguished by their level of integration with third party software, like collaboration, project management or issue tracking. Bitbucket (BITBUCKET) from Atlassian, for example offers integration with JIRA, Jenkins and Bamboo Continuous Integration servers as well as notification and chat services. CloudForge (CLOUDFORGE) by CollabNet integrates with TeamForge for collaboration, JIRA and Basecamp for project management. GitLab (GITLAB) offers, apart from JIRA connectivity a fine-grained workflow management integration.

GitLab and GitHub are the largest open source community and the industry-standard version control and publishing platforms for web developers. GitHub is a Git repository hosting service which offers integrated issue tracking, wikis and tools to enable collaborative code review and improvement. GitLab’s unique features include LDAP and two-factor user authentication.

Other key players include Microsoft’s CodePlex (CODEPLEX), with which users can create, share, collaborate and download from the project to the software phase, and Google’s Project Hosting on Google Code (GC), which provides a free collaborative development environment for open source projects. CodePlex has a source code control base on Mercurial, Team Foundation Server, Subversion or Git, project discussions, wiki pages, issue tracking and others. Project Hosting on the other hand has announced that it is reaching end-of-life on January 25th, 2016.

Another repository worth of mention is ProjectLocker’s (PL) and LaunchPad (LP). ProjectLocker’s has focused on safety and security features. It provides private enterprise-grade, Git or Subversion, source code repository hosting, featuring among others fine-grained directory-based permissions (Subversion), automatic backups of your data, bug tracking and wiki pages. LaunchPad uses the Bazaar version control system to host your project’s source code and it is able to build Ubuntu packages by using recipes directly from branches. It emphasizes cross-project collaboration and aims to be a front-end to all of open source. For that reason Launchpad is a centralized service rather than a product that users deploy on their own servers.

### Cloud SW modelling

Modelling tools refer to the applications used in order to describe the functional and nonfunctional requirements of a software development project. These tools usually include a designer for presenting the architecture of an information system, a process, an interface or a component. The most well-known language used by these tools is Unified Modelling Language (UML) (Fowler 2004). Most of the desktop IDEs have plugins for UML diagrams (like Rational Rose and BOUML), however there are open source state of the art tools like Argo UML (ARGOUML) and StarUML (STARUML), which have been widely used for desktop apps. Nevertheless, in the Cloud era the ability to create UML diagrams has been expanded through cloud modelling services, which are able to create any kind of diagrams.

The complexity and the variety of diagrams offered by the various tools differ. Simple UML, BPMN, Database designs and Flowcharts can be created by tools like Diagramo (DIAGRAMO), Gliffy (GLIFFY) or GenMyModel (GENMYMODEL), whereas more complicated models covering the phase of requirements gathering, process modelling, user experience prototyping and document production are offered by Visual Paradigm (VP). Creately (CREATELY) also offers a vast amount of diagram types, which cover various industries. It includes a built in mockup editor for UX design from desktop or mobile interfaces.

In terms of integration, visual-paradigm supports code engineering for a variety of platforms (Eclipse, Netbeans, Intellij.Net) and programming languages (C++, JAVA, C). It also offers a cloud repository called VPository, and enhances collaboration with some default tools like sharing diagrams and posting comments). In GenMyModel diagrams can be exported in multiple formats (jpg, xmi, svg, pdf) and imported by other software. Creately, on the other hand, has focused on integration, since it gives plugins for other platforms like Jira, Confluence, FogBugz, Google apps and Google store. It can export diagrams in pdf or SVG but it can also import Microsoft Visio files. Gliffy used to offer an REST API for developers who want to embed diagrams inside their own web applications, using PHP and JAVA libraries. However, since the beginning of 2015 the API is not updated and, as stated by the company, will not be supported.

Some of the aforementioned tools offer collaboration features among team members who are able to share their diagrams, view changes in real time and even chat with their colleagues with GenMyModel and Creately being the top in the this category.

Finally, it should be mentioned that although, all of these tools are offered as cloud services, Diagramo is an open source solution under a GPL license and can be installed on premises using a PHP web server and Creately can be downloaded as a desktop app.

### Cloud SW composition tools

The term ‘composition tools’ refers to development environments, which attempt to cover all phases of an applications coding lifecycle. These phases include editing, compiling, debugging, linking, testing and maintaining the application’s source code. These tools are often referred to as Integrated Development Environments—IDEs) and they have had a great acceptance in desktop applications because of their user friendly interfaces and intelligent code management (live syntax debugging, code optimization, ready UI components and libraries).

Migrating to the cloud has been proven a difficult task for most of these platforms. The basic reason is that their architecture was not initially designed for cloud applications. This means that their interface was implemented in a certain programming language (e.g. JAVA, C++ etc.) and it cannot be easily converted to a web based programming language, which is required by cloud applications. As a result, there is a new for new composition tools dedicated to cloud application development. On the other hand, we see that modern cloud programming environments have not managed to provide a complete solution on composition tools and they offer in many cases integration with the already existing desktop IDEs, using external plugins, which just upload the built project to the cloud servers.

Some of the most commonly used desktop IDEs are: Visual Studio, Eclipse and Netbeans.

Visual Studio (VS) is Microsoft’s IDE for developing Window’s desktop applications, web services, web sites and web applications, based on C, C++, C#, VB.NET, F#, M. Python, Ruby, Javascript, HTML and CSS. Its editor offers code completion and code refactoring and its debugger can be used both at source and machine level. It also provides design tools for forms, web services, database schemas and classes.

Eclipse (ECLIPSE) is an IDE mostly written in JAVA, which supports a variety of programming languages like Ada, ABAP, C, C++, COBOL Fortran, Haskell, JavaScript, Lasso, Lua, Natural, Perl, PHP, Prolog, Python, R, Ruby (including Ruby on Rails framework), Scala, Clojure, Groovy, Scheme, and Erlang. It is an open source platform, which offers its own SDK (Eclipse SDK) targeted for java developers and its own license (Eclipse Public license). Its architecture is based on a plug in model, which lets the platform to be extended using any programming language.

Netbeans (NETBEANS) follows the same philosophy like Eclipse. It is also written on JAVA, basically targeting JAVA programmers, however it can be used for PHP, C, C++, JavaScript and HTML projects. Apart from the basic programming tools, it incorporates a framework for developing Java Swing applications and an architecture which lets the platform be updated dynamically using modules stored in various repositories.

As reviewed in 3a, only Eclipse has managed to find a way for online integration though the Orion project. Most programming environments offer only certain parts of an IDE (basic editors and compilers) or integrated with existing desktop solutions.

### Cloud SW processing and documentation tools

Processing and documentation software tools cover the need of having an integrated hub of help and reference information. The variety of software within organizations make it difficult, if not impossible, to handle them efficiently. These tools provide advanced search filters, offer a single knowledge database pool and also enhance collaboration within teams.

The basic features offered by these tools include the ability to use WYSIWYG editors, HTML5, CSS and Javascript in order to create and publish help related content (user manuals, FAQs, context help etc.) and the ability to search inside the produced content in a user friendly and efficient way. Robohelp (RH), a Help Authoring Tool (HAT) by Adobe, has managed to render the information easily searchable and accessible with the use of dynamic filters and conditional tags. On the other hand, tools like ClickHelp (CLICKHELP) and HelpServer (HS) emphasize team collaboration and simplicity in terms of document editing, through role-based permissions, template creation and versioning. In terms of document formatting and integration with other systems, Helpiq (HELPIQ) is a clear winner, offering plenty of integrations with popular 3-party software (SnapEngage, Salesforce Desk.com, Google Translate, Wufoo Forms, etc.). Robohelp integrates with Dropbox and has CHM import functionality, a feature also seen in ClickHelp. All of them are cloud based and can be accessed by mobile devices, using modern responsive designs, with ClickHelp also providing desktop applications for Windows, Mac OS X and Linux.

### Cloud SW management and orchestration tools

Elasticity (Herbst et al. 2013) and scalability (Wu et al. 2009) are two of the main advantages of the Cloud Technology. However, flexibility in resources depending on live demand is an operation that requires constant monitoring of the executed Cloud apps. For this reason, the Cloud management and orchestration tools have been developed.

Big IaaS players, like Rackspace (RACKSPACE), offer infrastructure management and monitoring of the VMs inside their data centers. Some of the features include remote monitoring tests connectivity from regional zones deployed throughout Rackspace’s global data centers, and agent-based monitoring gathering information from inside each resource. They provide real-time alerts and notifications, and they are configurable and easy to set-up.

On the other hand, VMware, a leader in virtualization provides software monitoring with vRealize Hyperic (HYPERIC) (Rahabok 2014). It monitors operating systems, middleware and applications running in physical, virtual and cloud environments. It features monitoring configuration templates, comprehensive events analysis with predefined KPIs, custom UI, role-based notification system and escalation workflows.

One of the biggest player in this sector is probably Microsoft, who managed to integrate Azure (AZURE), its open, flexible, enterprise-grade cloud computing platform offering Iaas and PaaS, with monitoring solutions for infrastructure and software through its Azure Preview Portal. Event hubs, predictive analytics, schedulers, automations, operational insights (machine data) and a key vault are some of the portal’s main tools for monitoring one’s Cloud in Azure.

Apart from monitoring, there are also Cloud Portofolio Management tools, like Scalr (SCALR) (Aristotle 2012) and RightScale (RS), which try to go one step further by providing scalability among cloud platforms. They are both WWW based and they target in reducing the application delivery time and improve cost effectiveness. Scalr, concentrates on infrastructure security with its Orchestration Engine, Reusable Roles, High Level UI API and various integrations. It enforces infrastructure security with its Governance Compliance and Network Policy Enforcement. RightScale supports multi-clouds and hybrid clouds, while Scalr is also available as an on premise solution.

Other popular platforms offer embedded monitoring tools in their products i.e. IBM Bluemix Monitoring & Analytics (BLUEMIX), SAP HANA Advanced Analytics (HANA) etc. Lastly, there are quite a few open source cloud monitoring tools such as Ganglia (GANGLIA), Nagios (NAGIOS) and Zabbix (ZABBIX).

## Comparisons

Gradually, Cloud Programming Environments extended their offered services and overlapped functionalities that were initially provided by other categories. As such, we will focus below on the comparison of Cloud Programming Environments, as they are the most prominent Cloud Software category. As it happens with any new technology, businesses hesitate to move to the Cloud. This inertness has led many Cloud software providers to offer also On-Premise versions of their Cloud solutions as well as hybrid implementation options. Therefore, the comparison will also contain an extra criterion that of implementation.

All reviewed cloud programming environments (see comparison in Table 1) offer a code source code editor with syntax highlighting and code suggestions, depending on the available programming languages. Cloud9 uses a version of the well known ACE editor, available under a New BSD license and offering a look and feel closer to Microsoft Visual Studio. Orion, on the other hand, has incorporated the style of Eclipse editor, but it supports a narrower range of web browsers. Codeanywhere and Codenvy seem to have the most complete source code editors, supporting various browsers and different versions.

**Table 1:** Comparison of cloud programming environments

PPlatform	Modelling	IDEs					Documentation	Repository		Mobility	Implementation	
	Modelling	Editor	Debugging	Runtime auditing	Project upload	Database	Documentation	Source version control	External repository	Mobility	Cloud	On-premise
Compilr	–	V	–	–	V	–	–	–	V (GitHub)	–	V	–
jsFiddle	–	V	–	–	–	–	–	–	–	–	V	–
Cloud9	–	V	V (*node.js)	–	V	V (MySQL, MongoDB)	–	–	V (GitHub)	–	V	–
Codenvy	–	V	V (*docker files)	V	V	V (*)	–	–	V (Git, GitHub, BitBucket)	–	V	V
Eclipse Orion	–	V	–	–	–	–	–	–	V (Git)	–	V	V (*Eclipse)
Koding	–	V	–	–	V	V (*)	–	–	V (Git)	–	V	–
Codeanywhere	–	V	–	V	V (DropBox, FTP)	V (MySQL)	–	–	V (SVN, GitHub, Git)	–	V	–

Asterisks denote the partial implementation of a feature. Cloud 9 offers debugging only for Node.js programming, while Codenvy has it only using Docker files. In terms of Database, Codenvy and Koding let the developers install whatever they want on the VMs, so indirectly they offer this feature. Finally, Eclipse Orion, is a cloud version of Eclipse platform, which can be seen as its on premise equivalent

Debugging and runtime auditing features are yet in early stages. Only Cloud9 and Codenvy have debugging capabilities, the first one for server side javacript, using Node.js and the second one using prebuilt docker files, which must have been provided by third parties for the given programming languages. JsFiddle also has debugging functionalities since it is based on client executed code (Javascript). Codenvy and Codeanywhere offer some monitoring tools for real time auditing of the applications, such as a representation of the execution log or instant preview of the developer’s code, however, in most cases, developers have to write their own debugging and auditing tools, which renders cloud programming less efficient.

None of them provide an internal source code version control system, but rely on external repositories, with GIT and GitHub being the most widely used. Codeanywhere, Codenvy and Cloud9 seem to offer more solutions, including Bitbucket and SVN. As the competition grows, all programming environments try to adapt to new players in the repository market and new features like code comparison between different code versions.

As cloud IDEs all of them are offered as a service, whereas only Codenvy has an on-premise plan. Eclipse Orion can also be used on-premises but it is mostly based on the existing Eclipse IDE for desktop applications. Cloud9 and Codeanywhere have tried to give access to their developers through mobile devices apart from web browsers. This effort does not seems to have a wide support from the community, mostly because it is very difficult to incorporate coding functions in a limited screen of a mobile or tablet.

In terms of internal database support and installation, Cloud9 and Codeanywhere let the developers configure their MySQL or mongo databases, offering detailed manuals. Codenvy relies on docker files for database support and has a well designed wizard which helps the developers setup their data sources or connect to external ones. It also provides a simple SQL editor to test the connection and the SQL queries. Finally, Koding provides a VM, so the developer can use SSH to download and install MySQL manually.

Most of these IDEs have project-file upload functionalities, with the most complete solution offered by Codeanywhere. Apart from ftp client upload, connectivity with Dropbox and Google drive, which makes code synchronization among devices relatively easy, it offers the ability to share certain parts of the project with external contributors just by sending them a link. Cloud9 has focused on real time collaboration, using collaborative editing features with a feeling of Google docs.

Finally, current Cloud IDEs do not seem to offer solution for the design phase of a development project, which would require modeling tools for class or process designing. A productive programming environment should include UML editors, a wide range of diagrams, database designers or even documentation extractors, in order to facilitate the development as a whole process and not as a separate coding phase.

## Challenges and open issues

Software engineering in the cloud era has made its initial steps showing potential to provide concrete implementation and execution environments for cloud-based applications. However, a number of important challenges need to be addressed for this approach to be viable. We enumerate a few of them:

### Software engineering aspects

As it was made evident from the analysis of the previous chapters, although current solutions for cloud-based IDEs provide important new features and capabilities, they employ a subset of the features present in industrial-level desktop based environments. In this way they are able to address a large number of development needs, focusing on specific languages and application demands, but the integration of the full set of capabilities and flexibility existing in their desktop counterparts needs to be gradually incorporated. Debugging and runtime auditing needs to be further supported while a full set of languages and components (e.g. databases) need to be made available to the developer. This requires a substantial effort to incorporate functionality that is available to the developers for more than a decade. Moreover, increased and unpredicted latencies in the development process that cannot be easily managed by the environment itself as they depend on the network capabilities (Jackson et al. 2010) can cause significant problems in the development process. Critically, although cloud based environments are able to deliver extreme throughput for the compilation and testing of large-scale projects by employing large farms of compute nodes, small scale, infant projects may suffer from large latencies, a fact that can frustrate developers and discourage them from migrating their working environment to the cloud. Hybrid solutions, where local environments cooperate with cloud IDEs could provide an initial starting point to tackle the severe issues of latency.

Moreover, current solutions have partially taken advantage of the cloud capabilities to leverage collaboration (Graham 2011) and thus software productivity. Code sharing and versioning are absolute prerequisites, but they do not progress productivity beyond what is currently supported in traditional environments, nor do they make use of the advantages provided by cloud environments. Collaboration among development teams can be dramatically enhanced by multilayer programming, i.e. by developing components in different layers at the same time on the same project.

Furthermore, current cloud-based software engineering environments follow the traditional trends in application development. However, with object-oriented techniques having reached a point of exhaustion, Model-Driven Engineering (MDE) constitutes the latest paradigm shift in software engineering, as it raises the level of abstraction beyond that provided by 3rd generation programming languages. MDE decomposes system design and operational logic from implementation details by utilizing appropriate abstractions expressed as models. This decomposition greatly simplifies software development and is able to automate substantial parts of the process.

Cloud development has transformed in a competition of integrations with other languages and software, leaving outside the basic essence of the cloud idea, which is the ability to design, implement, test and deploy an application directly to the cloud. Until now there is a tendency to either create an environment with an editor and many embedded compilers/interpreters, or an environment where the development team can upload their desktop made code. Though this maybe a quite simple solution, one can easily understand that the first choice is not efficient and the second one is just not cloud implementation. A programming environment able to use multi-layer programming and MDE would offer the required levels of abstraction and would only need a simple programming language to glue things together. This may seem as a radical change, but we have seen it working in other domains.

### Interoperability

The existing landscape in cloud-based software development platforms has provided sufficient solutions for transferring a large number of applications to cloud infrastructures in a productive way. However, they are heavily based on ad-hoc solutions, which in several cases closely attach the developed applications to specific development environments and hosting infrastructures. Migrating projects from one platform to another or reusing components between platforms is by no means straightforward, as in several cases a number of the incorporated components are proprietary. The same holds for components that need to cooperate in order to deliver a higher level product. The situations becomes even more challenging in MDE based approaches that additionally incorporate higher level of concepts and tools like models, domain specific languages, and tools for automated model management (transformation, validation, comparison, merging, refactoring etc.). The use of open standards can provide a solid base for the development of interoperable modules, while basing the development on open-source components can minimize re-engineering efforts.

### Security

Data security is one of the most critical issues in cloud-based applications (Kaufman 2009; Sangroya et al. 2010; Jensen et al. 2009). The majority of users and enterprises are reluctant to trust sensitive data to cloud environments, and this is the main reason for the development of private clouds. Software is by no means an exception in this rule. Software projects are realized by large investments and constitute a critical capital of software engineering companies. Clearly, uploading source code to external environments is not an easy decision, even if this concerns code that will be finally released as open source, yet protected by one of the available licenses.

### Resource management

One of the key advantages of cloud computing is its ability to utilize centralized resources in order to deliver high quality services in a “pay as you go” fashion. In this way, it lowers costs by releasing users from the burden to invest on local infrastructure, while economy of scale enables additional benefits, as numerous small-scale and typically underutilized data centers are replaced by larger infrastructures that target higher resource efficiency. However, this creates a new challenge to manage resources in these large scale environments that host services with different characteristics, application demands and metrics for quality of service (Beloglazov and Buyya 2010; Delimitrou and Kozyrakis 2014; Younge et al. 2010). To get an idea on the challenges on the challenges involved, we may take a look at the power consumption relevant to data center operation hosting cloud infrastructures: Cloud operation is fast evolving as one of the most power-hungry human activities absorbing enormous and steadily increasing amounts of energy, with significant impact on the environment and the greenhouse emissions (Aravanis et al. 2015). In 2011, data center’s total energy consumption was around 271 billion kWh: enough to power up all residential households in industrialized countries such as UK or France, comparable to the total amount of energy consumed by Italy (Data Centre Dynamics 2011), or approximately 7 % of the US total energy consumption (Index Mundi 2011).

Based on this reality, advanced resource management engines need to be incorporated at all levels of a cloud ecosystem, from hardware, to cloud management software and up to the cloud applications themselves. Ideally, all these layers should collaborate in an efficient way to minimize resource consumption (a concern for cloud providers), without violating QoS as expressed in the relevant Service Level Agreements—SLAs (a concern for cloud customers). Thus, all cloud components including applications and application development environments would greatly benefit from intelligent management engines that are able to monitor resource consumption, analyze the current status, predict future demands, decide on more resource-efficient configurations and enforce/request those new configurations within the hosting cloud environment.

## Discussion and conclusions

In this paper we presented an overview of the state of the art platforms for cloud-based development. We reviewed the historical transformation of cloud development tools from simple code editors to modern programming environments, which are able to cover more than one stage in the development cycle. Most of these platforms focus on the programming stage, offering tools for a variety of languages, on the file version control, utilizing external repositories. On the other hand, the deployment of the produced applications seems to be in very early stages, based on the latest virtualization techniques and technologies. These technologies require specialized cloud system administration skills, which are not possessed by most development teams. Auditing and debugging are very difficult to control from a generalized platform, since each programming language offers different, cloud-unaware tools. Furthermore, the modeling tools required in the analysis stage are not included as part of the programming environments, not even as a part of integration with third party cloud modeling tools. The documentation stage suffers from similar problems.

The results of our review about cloud development are far from encouraging, since a compact, reliable solution does not yet exist. At the moment, only educational institutions are able to benefit from cloud development platforms, in order to facilitate students learn and adapt to programming languages and new technologies. Trying to explain this inability for cloud production development, we could say that till now the efforts have been focused on migrating the already existing desktop development technologies and methodologies to the cloud. The obvious advantage of this process is that developers would be able to write their code from anywhere in their favorite programming language directly on the cloud. However, it does not seem that this is the problem of development teams nowadays. Developers are used to write their code from a desktop or laptop and in reality it is much faster for them. Thus, a cloud programming environment should focus on the real problems, which are the inability to deploy the code in a production environment, to offer real time updates with zero shutdown time and to monitor the resources used. Furthermore, it is essential to provide features concerning the debugging and auditing of the application in a language and platform independent way and to have a consistent and reliable solution which will cover the development process as a whole and not only in one stage.

This re-targeting may mean that a programming environment should offer a totally new programming model, a new language, a new script, to which developers should adapt. It should be also taking into account that developers focus on results and they are not resistant to changes if the gains exceed expectations.

## References

[CR1] AKSHELL (2011) Akshell. https://github.com/akshell

[CR2] Ansari AN, Patil S, Navada A, Peshave A, Borole V (2011) Online C/C++ compiler using cloud computing. In: IEEE international conference on multimedia technology

[CR3] Anselmo D, Ledgard H (2003). Measuring productivity in the software industry. Commun ACM.

[CR4] ARGOUML. Argo UML http://argouml.tigris.org/

[CR5] Armbrust M, Fox A, Griffith R, Joseph AD, Katz R, Konwinski A, Zaharia M (2010). A view of cloud computing. Commun ACM.

[CR6] Aravanis AI, Velivassaki TH, Voulkidis AC, Zahariadis T, Cottis P (2015) Federated data centers as smart city stabilizing factors. In: 3rd international workshop on smart city and ubiquitous computing applications

[CR7] Aristotle J (2012) Scalr. Secut Press. ISBN:6139396824 9786139396825

[CR8] ASCI (2010) Artifact-centric service interoperation. www.acsi-project.eu

[CR9] AZURE. Microsoft Azure https://azure.microsoft.com

[CR10] Barroso LA, Clidaras J, Hölzle U (2013). The datacenter as a computer: an introduction to the design of warehouse-scale machines. Synt Lect Comput Archit.

[CR11] Beloglazov A, Buyya R (2010) Energy efficient resource management in virtualized cloud data centers. In: Proceedings of the 10th IEEE/ACM international conference on cluster, cloud and grid computing. IEEE Computer Society

[CR12] Beloglazov A, Abawajy J, Buyya R (2012). Energy-aware resource allocation heuristics for efficient management of data centers for cloud computing. Future Gener Comput Syst.

[CR13] Beydeda S, Book M, Gruhn V (2005). Model-driven software development.

[CR14] BITBUCKET. Bitbucket https://bitbucket.org/

[CR15] BLUEMIX. IBM Bluemix Monitoring & Analytics https://developer.ibm.com/bluemix/docs/category/monitoring-analytics

[CR16] Buyya R (2009). Cloud computing and emerging IT platforms: vision, hype, and reality for delivering computing as the 5th utility. Future Generation computer systems.

[CR17] CA. Codeanywhere https://codeanywhere.net/

[CR18] CIMI (2014) Cloud infrastructure management interface. http://dmtf.org/standards/cmwg

[CR19] Ciortea L, Zamfir C, Bucur S, Chipounov V, Candea G (2010). Cloud9: a software testing service. ACM SIGOPS Oper Syst Rev Arch.

[CR20] CLICKHELP. ClickHelp clickhelp.co

[CR21] CLOUD4SOA (2010) Cloud4SOA project. http://www.cloud4soa.eu

[CR22] CLOUD9. Cloud9 https://c9.io

[CR23] CLOUDFORGE. CloudForge http://cloudforge.com

[CR24] CODENVY. Codenvy https://codenvy.com

[CR25] CODEPLEX. CodePlex https://www.codeplex.com/

[CR26] CODERUN. Coderun http://www.coderun.com

[CR27] COMPILR. Compilr http://compilr.com

[CR28] CREATELY. Creately http://creately.com

[CR29] Data Centre Dynamics (2011) Global industry census 2011. http://www.dataCentredynamics.com/research/market-growth-2011-2012. Accessed 21 Sep 2015

[CR30] Delimitrou C, Kozyrakis C (2014). Quasar: resource-efficient and qos-aware cluster management. ACM SIGPLAN Not.

[CR31] DIAGRAMO. Diagramo http://diagramo.com

[CR32] ECLIPSE. Eclipse https://eclipse.org/ide

[CR33] Fowler M (2004). UML distilled: a brief guide to the standard object modeling language.

[CR34] GANGLIA. Ganglia scalable distributed monitoring system for high-performance computing systems http://ganglia.sourceforge.net

[CR35] GC. Google Code https://code.google.com/

[CR36] GENMYMODEL. GenMyModel https://www.genmymodel.com/

[CR37] GIT. Git https://git-scm.com

[CR38] GITLAB. GitLab https://about.gitlab.com

[CR39] GLIFFY. Gliffy https://www.gliffy.com

[CR40] Goldman M, Little G, Miller RC (2011) Real-time collaborative coding in a web IDE. In: Proceedings of the 24th annual ACM symposium on user interface software and technology, ACM, pp 155–164

[CR41] Graham M (2011). Cloud collaboration: peer-production and the engineering of the internet. Engineering earth.

[CR42] HANA. SAP HANA http://hana.sap.com

[CR43] Heineman GT, Councill WT (2001). Component-based software engineering. Putting the pieces together.

[CR44] HELPIQ. Helpiq http://www.helpiq.com/

[CR45] Herbst NR, Kounev S, Reussner R (2013) Elasticity in cloud computing: what it is, and what it is not. In: International conference on automatic computing, San Jose, CA. 26–28 June 2013

[CR46] Hobfeld T, Schatz R, Varela M, Timmerer C (2012). Challenges of QoE management for cloud applications. IEEE Commun Mag.

[CR47] HS. HelpServer http://www.helpserver.eu

[CR48] HYPERIC. vRealize Hyperic http://www.vmware.com/products/vrealize-hyperic

[CR49] Index Mundi (2011) Historical data graphs per year http://www.indexmundi.com/g/g.aspx?v=81&c=us&l=en. Accessed 21 Sep 2015

[CR50] Jackson KR et al (2010) Performance analysis of high performance computing applications on the amazon web services cloud. In: IEEE second international conference on cloud computing technology and science

[CR51] Jam J, Brannock E, Dekhane S (2010). JavaWIDE: innovation in an online IDE: tutorial presentation. J Comput Sci Coll.

[CR52] Jensen M, Schwenk J, Gruschka N, Iacono LL (2009) On technical security issues in cloud computing. In: IEEE international conference on cloud computing

[CR53] JSFIDDLE. jsFiddle https://jsfiddle.net

[CR54] Kats LCL et al (2012) Software development environments on the web: a research agenda. In: Proceedings of the ACM international symposium on New ideas, new paradigms, and reflections on programming and software. ACM

[CR55] Kaufman LM (2009). Data security in the world of cloud computing. IEEE Secur Priv.

[CR56] Kleppe AG (2003). MDA explained: the model driven architecture: practice and promise.

[CR57] KODING. Koding https://koding.com

[CR58] Lawrance J, Jung S (2013). Git on the cloud. J Comput Sci Coll.

[CR59] LP. LaunchPad https://launchpad.net/

[CR60] Mehta N, Gupta VK (2013) A survey on use of SaaS of cloud in education. In: International conference on cloud, big data and trust, Nov 13–15

[CR61] MERCURIAL. Mercurial https://mercurial.selenic.com/

[CR62] MONDO (2007) MONDO: scalable modeling and model management on the cloud. http://www.mondo-project.org

[CR63] NAGIOS. Nagios Monitoring System https://www.nagios.org

[CR64] NETBEANS. Netbeans https://netbeans.org

[CR65] OAUTH (2006) OAuth 2.0. http://oauth.net/2/

[CR66] OCCI (2010) Open Cloud Computing Interface. http://occi-wg.org/

[CR67] OID (2014) OpenID. http://openid.net/

[CR68] OVF (2014) Open Virtualization Format. http://www.dmtf.org/standards/ovf

[CR69] Piao JT, Yan J (2010) A network-aware virtual machine placement and migration approach in cloud computing. In: 9th IEEE international conference on grid and cooperative computing

[CR70] PL. ProjectLocker http://projectlocker.com/

[CR71] RACKSPACE. Rackspace Cloud Monitoring http://www.rackspace.com/cloud/monitoring

[CR72] Rahabok I (2014) VMware vRealize operations performance and capacity management, Packt Publishing, ISBN: 1783551682 9781783551682

[CR73] REMICS (2010) REuse and Migration of legacy applications to Interoperable Cloud Services. http://www.remics.eu

[CR74] RH. Robohelp http://www.adobe.com/RoboHelp‎

[CR75] RS. RightScale http://www.rightscale.com

[CR76] Sangroya A, Kumar S, Dhok J, Varma V (2010). Towards analyzing data security risks in cloud computing environments. Information Systems, Technology and Management.

[CR77] SCALR. Scalr Cloud Management Solves http://www.scalr.com

[CR78] Schmidt R (2012). Scalable business process enactment in cloud environments. Enterprise, business-process and information systems modeling.

[CR79] SF. SourceForge https://sourceforge.net

[CR80] Simmhan Y, VanIngen C, Subramanian G, Li J (2010) Bridging the gap between desktop and the cloud for escience applications. In: IEEE 3rd international conference on cloud computing

[CR81] SOAP (2007) Simple Object Access Protocol. http://www.w3.org/TR/soap/18273808

[CR82] Stahl T, Voelter M, Czarnecki K (2006). Model-driven software development: technology, engineering, management.

[CR83] STARUML. StarUML http://staruml.io/

[CR84] SUBVERSION. Subversion https://subversion.apache.org/

[CR85] Thomas E (2004). Service-oriented architecture: a field guide to integrating XML and web services.

[CR86] Voorsluys W, Broberg J, Buyya R, Buyya R, Broberg J, Goscinski A (2011). Introduction to cloud computing. Cloud computing: principles and paradigms.

[CR87] VP. Visual Paradigm http://www.visual-paradigm.com

[CR88] VS. Microsoft Visual Studio https://www.visualstudio.com

[CR89] Wu J, Liang Q, Bertino E (2009) Improving scalability of software cloud for composite web services. In: IEEE Conference on Cloud Computing, 2009. CLOUD ’09. IEEE, Bangalore, pp 143–146

[CR90] Younge AJ et al (2010) Efficient resource management for cloud computing environments. In: IEEE international green computing conference

[CR91] ZABBIX. Zabbix http://www.zabbix.com

[CR92] Zhu Y, Wang H, Hu Z, Ahn GJ, Hu H, Yau SS (2010). Efficient provable data possession for hybrid clouds. Proceedings of the 17th ACM Conference on Computer and Communications Security.

[CR93] Zissis D, Lekkas D (2012). Addressing cloud computing security issues. Future Gener Comput Syst.

